# Screening for breech presentation using universal late-pregnancy ultrasonography: A prospective cohort study and cost effectiveness analysis

**DOI:** 10.1371/journal.pmed.1002778

**Published:** 2019-04-16

**Authors:** David Wastlund, Alexandros A. Moraitis, Alison Dacey, Ulla Sovio, Edward C. F. Wilson, Gordon C. S. Smith

**Affiliations:** 1 Cambridge Centre for Health Services Research, Cambridge Institute of Public Health, Cambridge, United Kingdom; 2 The Primary Care Unit, Department of Public Health and Primary Care, University of Cambridge, Cambridge, United Kingdom; 3 Department of Obstetrics and Gynaecology, University of Cambridge, NIHR Cambridge Comprehensive Biomedical Research Centre, Cambridge, United Kingdom; 4 Health Economics Group, Norwich Medical School, University of East Anglia, Norwich, United Kingdom; University of Manchester, UNITED KINGDOM

## Abstract

**Background:**

Despite the relative ease with which breech presentation can be identified through ultrasound screening, the assessment of foetal presentation at term is often based on clinical examination only. Due to limitations in this approach, many women present in labour with an undiagnosed breech presentation, with increased risk of foetal morbidity and mortality. This study sought to determine the cost effectiveness of universal ultrasound scanning for breech presentation near term (36 weeks of gestational age [wkGA]) in nulliparous women.

**Methods and findings:**

The Pregnancy Outcome Prediction (POP) study was a prospective cohort study between January 14, 2008 and July 31, 2012, including 3,879 nulliparous women who attended for a research screening ultrasound examination at 36 wkGA. Foetal presentation was assessed and compared for the groups with and without a clinically indicated ultrasound. Where breech presentation was detected, an external cephalic version (ECV) was routinely offered. If the ECV was unsuccessful or not performed, the women were offered either planned cesarean section at 39 weeks or attempted vaginal breech delivery. To compare the likelihood of different mode of deliveries and associated long-term health outcomes for universal ultrasound to current practice, a probabilistic economic simulation model was constructed. Parameter values were obtained from the POP study, and costs were mainly obtained from the English National Health Service (NHS). One hundred seventy-nine out of 3,879 women (4.6%) were diagnosed with breech presentation at 36 weeks. For most women (96), there had been no prior suspicion of noncephalic presentation. ECV was attempted for 84 (46.9%) women and was successful in 12 (success rate: 14.3%). Overall, 19 of the 179 women delivered vaginally (10.6%), 110 delivered by elective cesarean section (ELCS) (61.5%) and 50 delivered by emergency cesarean section (EMCS) (27.9%). There were no women with undiagnosed breech presentation in labour in the entire cohort. On average, 40 scans were needed per detection of a previously undiagnosed breech presentation. The economic analysis indicated that, compared to current practice, universal late-pregnancy ultrasound would identify around 14,826 otherwise undiagnosed breech presentations across England annually. It would also reduce EMCS and vaginal breech deliveries by 0.7 and 1.0 percentage points, respectively: around 4,196 and 6,061 deliveries across England annually. Universal ultrasound would also prevent 7.89 neonatal mortalities annually. The strategy would be cost effective if foetal presentation could be assessed for £19.80 or less per woman. Limitations to this study included that foetal presentation was revealed to all women and that the health economic analysis may be altered by parity.

**Conclusions:**

According to our estimates, universal late pregnancy ultrasound in nulliparous women (1) would virtually eliminate undiagnosed breech presentation, (2) would be expected to reduce foetal mortality in breech presentation, and (3) would be cost effective if foetal presentation could be assessed for less than £19.80 per woman.

## Abstract 2

In their cohort study, David Wastlund and colleagues find that universal ultrasound scanning for breech presentation near term is associated with reduced undiagnosed breech presentation and improved pregnancy outcomes, and can be cost-effective.

## Author summary

**Why was this study done?:**

- Risks of complications at delivery are higher for babies that are in a breech position, but sometimes breech presentation is not discovered until the time of birth.
- Ultrasound screening could be used to detect breech presentation before birth and lower the risk of complications but would be associated with additional costs.
- It is uncertain if offering ultrasound screening to every pregnancy is cost effective.

**What did the researchers do and find?:**

- This study recorded the birth outcomes of pregnancies that were all screened using ultrasound.
- Economic modelling and simulation was used to compare these outcomes with those if ultrasound screening had not been used.
- Modelling demonstrated that ultrasound screening would lower the risk of breech delivery and, as a result, reduce emergency cesarean sections and the baby’s risk of death.

**What do these findings mean?:**

- Offering ultrasound screening to every pregnancy would improve the health of mothers and babies nationwide.
- Whether the health improvements are enough to justify the increased cost of ultrasound screening is still uncertain, mainly because the cost of ultrasound screening for presentation alone is unknown.
- If ultrasound screening could be provided sufficiently inexpensively, for example, by being used during standard midwife appointments, routinely offering ultrasound screening would be worthwhile.

## Introduction

Undiagnosed breech presentation in labour increases the risk of perinatal morbidity and mortality and represents a challenge for obstetric management. The incidence of breech presentation at term is around 3%–4% [1–3], and fewer than 10% of foetuses who are breech at term revert spontaneously to a vertex presentation [4]. Although breech presentation is easy to detect through ultrasound screening, many women go into labour with an undetected breech presentation [5]. The majority of these women will deliver through emergency cesarean section (EMCS), which has high costs and increased risk of morbidity and mortality for both mother and child.

In current practice, foetal presentation is routinely assessed by palpation of the maternal abdomen by a midwife, obstetrician, or general practitioner. The sensitivity of abdominal palpation varies between studies (range: 57%–70%) and depends on the skill and experience of the practitioner [6,7]. There is currently no guidance on what is considered an acceptable false negative rate when screening for breech presentation using abdominal palpation. In contrast, ultrasound examination provides a quick and safe method of accurately identifying foetal presentation.

Effective interventions exist for the care of women who have breech presentation diagnosed near term. The Royal College of Obstetricians and Gynaecologists recommends ‘that all women with an uncomplicated breech presentation at term should be offered external cephalic version (ECV)’ [2]. The rationale for this is to reduce the incidence of breech presentation at term and avoid the risks of vaginal breech birth or cesarean section. The success rate of ECV is considered to be approximately 50% [2,8,9], but it differs greatly between nulliparous and parous women (34% and 66%, respectively) [9]. ECV is overall safe, with less than 1% risk to the foetus and even smaller risk to the mother [10]; despite this, a significant number of women decline ECV for various reasons [11]. Should ECV be declined or fail, generally women are offered delivery by planned (elective) cesarean section, as there is level 1 evidence of reduced risk of perinatal death and severe morbidity compared with attempting vaginal breech birth, and it is also associated with lower costs [3,12,13]. However, some women may still opt for an attempt at vaginal breech birth if they prioritise nonintervention over managing the relatively small absolute risks of a severe adverse event [1,14].

We sought to assess the cost effectiveness of universal late-pregnancy ultrasound presentation scans for nulliparous women. We used data from the Pregnancy Outcome Prediction (POP) study, a prospective cohort study of >4,000 nulliparous women, which included an ultrasound scan at 36 weeks of gestational age (wkGA) [15]. Here, we report the outcomes for pregnant nulliparous women with breech presentation in the study and use these data to perform a cost effectiveness analysis of universal ultrasound as a screening test for breech presentation.

## Methods

### Study design

The POP study was a prospective cohort study of nulliparous women conducted at the Rosie Hospital, Cambridge (United Kingdom) between January 14, 2008 and July 31, 2012, and the study has been described in detail elsewhere [15–17]. Ethical approval for the study was obtained from the Cambridgeshire 2 Research Ethics Committee (reference 07/H0308/163), and all participants provided informed consent in writing. Participation in the POP study involved serial phlebotomy and ultrasound at approximately 12 wkGA, 20 wkGA, 28 wkGA, and 36 wkGA [16]. The outcome of pregnancy was obtained by individual review of all case records by research midwives and by linkage to the hospital’s electronic databases of ultrasonography, biochemical testing, delivery data, and neonatal care data. The research ultrasound at 36 wkGA was performed by sonographers and included presentation, biometry, uteroplacental Doppler, and placental location. The ultrasound findings were blinded except in cases of breech presentation, low lying placenta, or foetal concerns such as newly diagnosed foetal anomaly and an amniotic fluid index (AFI) < 5 cm. This study was not prospectively defined in the POP study protocol paper [16] but required no further data collection.

If the foetus was in a breech presentation at 36 wkGA, women were counselled by a member of the medical team. In line with guidelines from the National Institute for Health and Care Excellence (NICE), ECV was routinely offered unless there was a clinical indication that contraindicated the procedure, e.g., reduced AFI (<5 cm) [18]. ECV was performed by 1 of 5 obstetric consultants in the unit between 36–38 wkGA, patients were scanned before the procedure to confirm presentation, and it was performed with ultrasound assessment; 0.25 mg terbutaline SC was given prior to the procedure at the discretion of the clinician. If women refused ECV or the procedure failed, the options of vaginal breech delivery and elective cesarean section (ELCS) were discussed and documented. The local guideline for management of breech presentation, including selection criteria for vaginal breech delivery, was based upon recommendations from the Royal College of Obstetricians and Gynaecologists (RCOG) [1]. We extracted information about ECV from case records that were individually reviewed by research midwives. Finally, we obtained delivery-related information from our hospital electronic database (Protos; iSoft, Banbury, UK).

Foetal outcomes included mode of delivery (MOD), birth weight, and gestational age at delivery. We used the UK population reference for birthweight, with the 10th and 90th percentile cut-offs for small and large for gestational age, respectively; the centiles were adjusted for sex and gestational age [19]. Maternal age was defined as age at recruitment. Smoking status, racial ancestry, alcohol consumption, and BMI were taken from data recorded at the booking assessment by the community midwife. Socioeconomic status was quantified using the Index of Multiple Deprivation (IMD) 2007, which is based on census data from the area in the mother’s postcode [20]. Ethical approval for the study was obtained from the Cambridgeshire 2 Research Ethics Committee (reference 07/H0308/163), and all participants provided informed consent in writing.

This study is reported as per the Strengthening the Reporting of Observational Studies in Epidemiology (STROBE) guideline.

### Statistical analysis

Data are presented as median (interquartile range) or *n* (%), as appropriate. *P* values are reported for the difference between groups calculated using the two-sample Wilcox rank-sum (Mann–Whitney) test for continuous variables and the Pearson Chi-square test for categorical variables, with trend tests when appropriate. Comparisons were performed using Stata (version 15.1). Missing values were included in the presentation of patient characteristics and outcomes but were excluded from the economic analysis and estimation of parameters.

### Economic model and analysis

To evaluate the cost effectiveness of routinely offering late-pregnancy presentation scans, a decision-tree simulation model was constructed using R (version 3.4.1) [21–24]. The time horizon of the economic analysis was from the ultrasound scan (36 wkGA) to infant lifetime, and costs were from the perspective of the English National Health Service (NHS). Costs for modes of delivery were obtained from NHS reference costs [25]; since these do not list a separate cost for vaginal breech delivery, we assumed that the cost ratio between vaginal breech and ELCS deliveries was the same as in another study (see Supporting information, S1 Text) [12].

**Fig 1:**
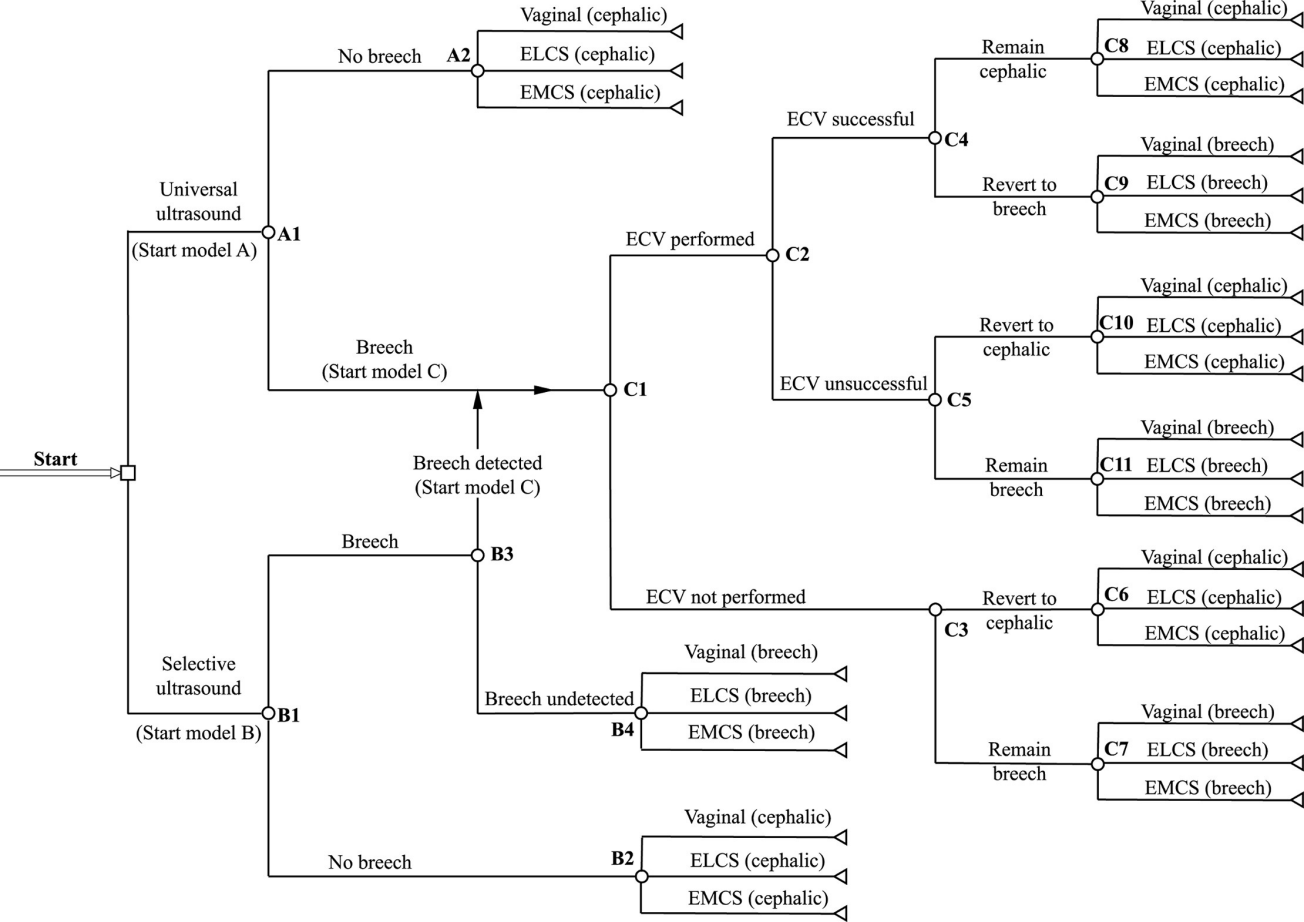
Simulation model structure. Structure of economic simulation model. ‘Universal ultrasound’ strategy starts in Model A, and patients with breech presentation enter Model C. ‘Selective ultrasound’, i.e., no routine ultrasound, starts in Model B, and only those with a detected breech presentation enter Model C. The letter–number codes for each node are equivalent to the codes in Table 1. ELCS, elective cesarean section; EMCS, emergency cesarean section.

The population of interest is unselected nulliparous women. The model compares the outcomes at birth for two strategies: ‘universal ultrasound’ and ‘selective ultrasound’ (Fig 1). For universal ultrasound, we assumed that all breech presentations at the time of scanning would be detected (i.e., assumed 100% sensitivity and specificity for the test). For selective ultrasound, the breech presentation was diagnosed either clinically (by abdominal palpation followed by ultrasound for confirmation) or as an incidental finding during a scan for a different indication. These assumptions were based upon current practice and derived from the POP study.

Compared to a standard antenatal ultrasound for which, typically, multiple measurements are made, an ultrasound scan for foetal presentation alone is technically simple. We theorised that such a scan could be provided by an attending midwife in conjunction with a standard antenatal visit in primary care, using basic ultrasound equipment. Since a specific unit cost for a scan for foetal presentation alone is not included in the national schedule of reference costs [25], we estimated the cost of ultrasound to include the midwife’s time, the cost of equipment, and room. More details are presented in the Supporting information, S1 Text. The cost of ECV was obtained from James and colleagues [26] and converted to the 2017 price level using the Hospital and Community Health Services (HCHS) index [27]. The probability of ECV uptake and success rate as well as MOD were obtained from the POP study. All model inputs are presented in Table 1 and S1 Table, and the calculation of cost inputs is shown in Supporting information, S1 Text.

**Table 1:** Inputs for costs and probabilities for the economic model.

**Costs**	**Costs**	**Source**				
Ultrasound scanning	20.7	Expert opinion*				
ECV	297.4	James et al. (2001) [26] †				
CV delivery	2,297.3	NHS Reference costs 2015–16 [25] ‡				
Elective cesarean delivery	3,438.1	NHS Reference costs 2015–16 [25] ‡				
Emergency cesarean delivery	4,553.4	NHS Reference costs 2015–16 [25] ‡				
VB delivery	3,999.7	Expert opinion*				
**Probabilities**	**Alpha**	**Beta**	**Mean**	**Node**	**Source**	
Breech prevalence at approximately 36 wkGA	179	3,700	0.046	A1 and B1	POP study	
ECV attempted	84	93	0.475	C1	POP study	
Detection without ultrasound	79	96	0.451	B3	POP study	
Successful ECV	12	72	0.143	C2	POP study	
SRC (ECV not attempted)	21	72	0.226	C3	POP study	
SRB	1	11	0.083	C4	POP study	
SRC (failed ECV)	3	127	0.023	C5	Ben-Meir and colleagues [28]§	
**MOD**	**CV**	**ELCS**	**EMCS**	**VB**	**Node**	**Source**
No breech	2,813	141	735	0	A2 and B2	POP study
Cephalic (successful ECV)	8	0	3	0	C8	POP study
Cephalic (spontaneous reversion)	11	1	9	0	C6 and C10	POP study
Breech (ECV not attempted)	0	52	20	0	C7	POP study
Breech (unsuccessful ECV)	0	54	18	0	C11	POP study
Breech (spontaneous reversion)	0	0	15	11	C9	Leung and colleagues [5]
Undetected breech	0	0	15	11	B4	Leung and colleagues [5]

**Abbreviations:** CV, cephalic vaginal; ELCS, elective cesarean section; EMCS, emergency cesarean section; MOD, mode of delivery; NHS, National Health Service; POP, Pregnancy Outcome Prediction; SRB, spontaneous reversion to breech; SRC, spontaneous reversion to cephalic; VB, vaginal breech.

Costs given per unit/episode. For probabilities, alpha represent case of event and beta case of no event. MOD shows input values for Dirichlet distribution. Node refers to the chance nodes in Fig 1.

*Details on how this value was estimated is provided as Supporting information, S1 Text.

†Cost for ECV (high staff cost), converted to 2017 price level using the HCHS index [27].

‡Weighted average of all complication levels (Total HRGs).

§Due to the small sample size for these parameters in the POP study, the model used inputs for MOD for undetected breech instead.

The end state of the decision tree was the MOD, which was either vaginal, ELCS, or EMCS. Delivery could be either cephalic or breech. EMCS could be either due to previously undiagnosed breech presentation or for other reasons. All cases of breech could spontaneously revert to cephalic presentation. However, we assumed the probability of this to be lower if ECV had been attempted and failed [28]. If ECV was successful, a reversion back to breech presentation was possible. It is currently unclear whether the probability of MOD varies depending on whether cephalic presentation is the result of successful ECV or spontaneous reversion [2,10,29–31], but we assumed that the probabilities differed.

Long-term health outcomes were modelled based upon the mortality risk associated with each MOD. The risk of neonatal mortality was taken from the RCOG guidelines. For breech presentation, these risks were 0.05% for delivery through ELCS and 0.20% for vaginal delivery. The risk of neonatal mortality for cephalic presentation with vaginal delivery was 0.10% [1]. There were no randomised clinical trials that allowed us to compare the outcomes of ELCS versus vaginal delivery for uncomplicated pregnancies with cephalic presentation; however, most observational studies found no significant difference in neonatal mortality and serious morbidity between the two modes [32–34]. For this reason, we assumed the mortality risk for cephalic vaginal and ELCS deliveries to be identical. We also assumed that EMCS would have the same mortality rate as ELCS, both for cephalic and breech deliveries. Studies have found that the MOD for breech presentation affects the risk of serious neonatal morbidity in the short term but not in the long term [1,3,35]. For this reason, we focused the economic analysis on the effect from mortality only. The average lifetime quality-adjusted life-years (QALYs) per member of the UK population was estimated using data on quality of life from Euroqol, weighted by longevity indexes from the Office for National Statistics (ONS) [36,37]. Using the annual discount rate of 3.5%, as recommended by NICE, the net present value for the average lifetime QALYs at birth was 24.3 [38].

The model was probabilistic, capturing how uncertainty in the input parameters affected the outputs by allowing each parameter to vary according to its distribution. Binary and multivariable outcomes were modelled using the beta and the Dirichlet distributions, respectively [39]. Probabilities of events were calculated from the POP study and presented in Table 1. On top of the probabilistic sensitivity analysis (PSA), the sensitivity of individual parameters was also explored through one-way sensitivity analyses modifying probabilities by +/− 1 percentage point and costs by +/− £10 to see which parameters had the greatest impact on cost effectiveness estimates.

Total costs depended on the distribution of MOD, the number of expected mortalities, and the cost of ultrasound scanning and ECV. Nationwide costs for each screening strategy were calculated for 585,489 deliveries, i.e., the number of births in England from 2016–2017, assuming 92% occur after 36 wkGA [15,40]. Model parameters were sampled from their respective distributions in a PSA of 100,000 simulations for each strategy. To determine cost effectiveness, we used two different willingness-to-pay thresholds: £20,000 and £30,000 [38]. A copy of the model code is available from the corresponding author (EW) upon request.

## Results

**Fig 2:**
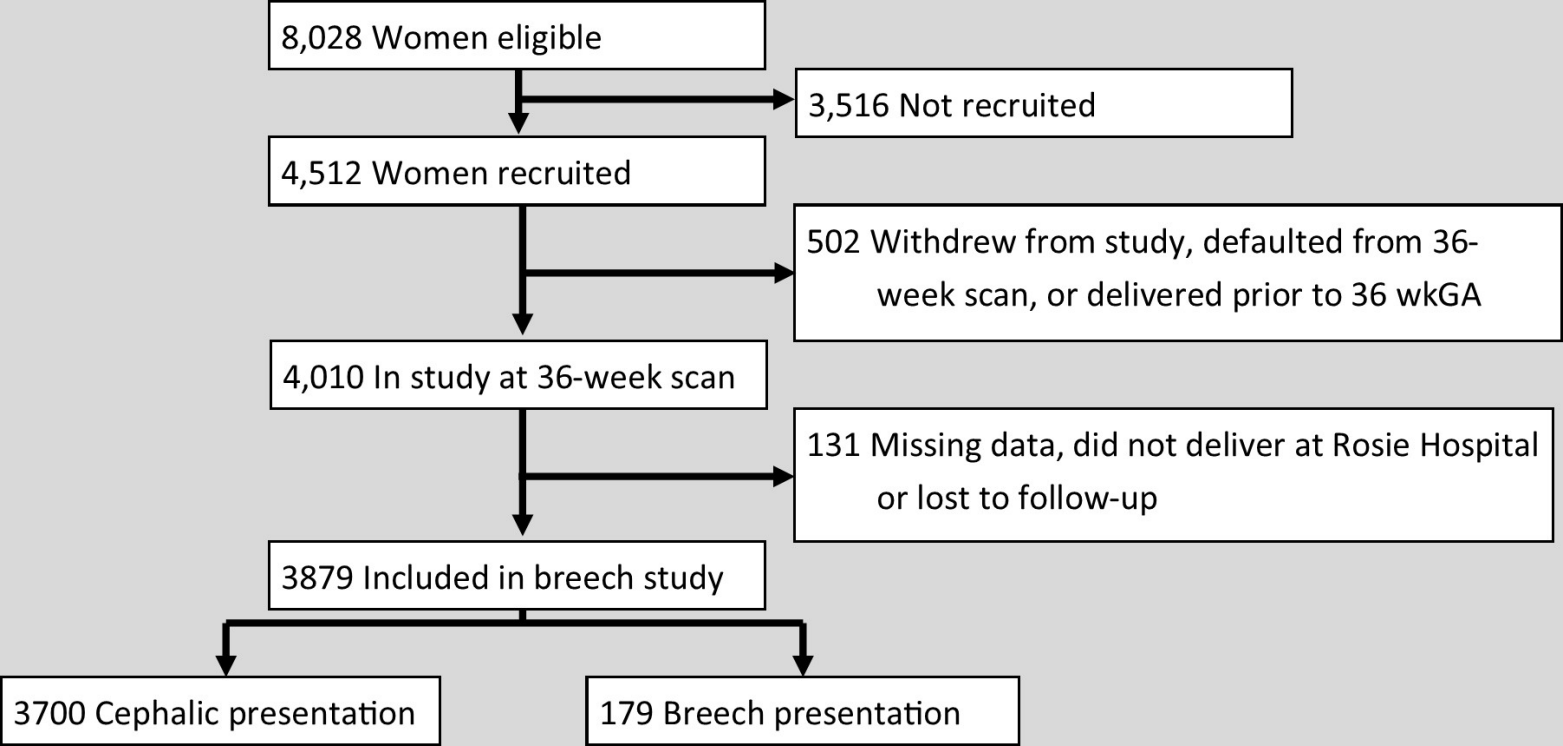
Patient recruitment. Schedule of patient recruitment in the POP study shown by foetal presentation. POP, Pregnancy Outcome Prediction.

Recruitment to the POP study cohort is shown in Fig 2 and has been previously described [17]. Information about presentation at the 36-week scan was available for 3,879 women who delivered at the Rosie Hospital, Cambridge, UK; 179 of these had a breech presentation.

We compared maternal and foetal characteristics of the 179 women with breech presentation at 36 weeks to the women with a cephalic presentation (Table 2). Women diagnosed with breech presentation were, on average, a year older than women with a cephalic presentation, but other maternal characteristics did not differ. The babies of women diagnosed breech were smaller and born earlier, but their birth weight centile and the proportions of small for gestational age (SGA) or large for gestational age (LGA) were not markedly different. There were no differences in maternal BMI between the groups. As expected, women with breech presentation were more likely to deliver by ELCS or EMCS.

**Table 2:** Characteristics and delivery outcomes in the POP study by presentation at 36 weeks.

Characteristics	Breech (*N* = 179)	Cephalic (*N* = 3,700)	*P* value
**Maternal**			
Age (years)	31 (28–34)	30 (27–33)	0.002
Age stopped FTE (years)	21 (18–23)	21 (18–23)	0.19
Missing	5 (3%)	105 (3%)	
Racial ancestry			
White European	172 (96%)	3,437 (93%)	0.38
Missing	0 (0%)	66 (2%)	
Alcohol consumption	7 (4%)	172 (5%)	0.65
Missing	0 (0%)	1 (<0.1%)	
Smoker	4 (2%)	179 (5%)	0.11
BMI, kg/m^2^	24 (22–27)	24 (22–27)	0.69
Missing	0 (0%)	1 (<0.1%)	
Deprivation quartile			0.08
1 (lowest)	46 (26%)	899 (24%)	
2	53 (30%)	873 (24%)	
3	39 (22%)	886 (24%)	
4 (highest)	33 (18%)	892 (24%)	
Missing	8 (4%)	150 (4%)	
**Foetal or neonatal**			
Female sex	96 (54%)	1,841 (50%)	0.31
Missing	0 (0%)	1 (<0.1%)	
Birth weight (grams)	3,310 (2,995–3,560)	3,445 (3,145–3,750)	<0.001
Gestational age (weeks)	39.1 (38.7–39.7)	40.4 (39.4–41.3)	<0.001
Birth weight centile	49 (25–70)	44 (24–66)	0.22
Birth weight centile category			0.32
SGA	12 (7%)	332 (9%)	
AGA	158 (88%)	3,199 (86%)	
LGA	9 (5%)	168 (5%)	
Missing	0 (0%)	1 (<0.1%)	
**MOD**			<0.001
Spontaneous vaginal cephalic	11 (6.1%)	1,885 (50.9%)	
Instrumental vaginal cephalic	8 (4.5%)	928 (25.1%)	
Elective cesarean section	110 (61.5%)	141 (3.8%)	
Emergency cesarean section	50 (27.9%)	735 (19.9%)	
Missing	0 (0%)	11 (0.3%)	

**Abbreviations:** AGA, appropriate for gestational age; FTE, full-time education; LGA, large for gestational age; MOD, mode of delivery; POP, Pregnancy Outcome Prediction; SGA, small for gestational age.

Statistics are presented as *n* (%) for binary outcomes and median (interquartile range) for continuous variables. The "Missing" category was not included in statistical tests. For variables without a "Missing" category, data were 100% complete. *P* values are reported for the difference between groups using the two-sample Wilcox rank-sum test for continuous variables and the Pearson Chi-square test for categorical variables, with trend test as appropriate (i.e., for deprivation quartile and birth weight centile category).

Breech presentation was suspected before the 36-wkGA scan for 79 (44.1%) of the women with breech presentation through abdominal palpation by the midwife or doctor; out of these, 27 had a clinically indicated scan between 32–36 weeks in which the presentation was reported. For 96 women, the breech presentation was unsuspected before the 36-week scan. Information on suspected breech position was missing for 4 women. There were no differences in BMI between the 79 women with suspected breech and the 96 women misdiagnosed as cephalic prior to the scan (median BMI was 24 in both groups, Wilcoxon rank-sum test *P* = 0.31).

MOD by ECV status is shown in Table 3. ECV was performed for 84 women, declined by 45 women, and unsuitable for 23; contraindications included low AFI at screening (18 women), uterine abnormalities (2), and other reasons (3). For 25 women, an ECV was never performed despite consent; 17 babies turned spontaneously, 6 had reduced AFI on the day of the ECV, and 2 went into labour before ECV. When performed, ECV was successful for 12 women; in one case, the baby later reverted to breech presentation before delivery. Information on ECV uptake was missing for 2 women. Foetal presentation and ECV status in the structure of the economic model is shown in Supporting information, S1 Fig.

**Table 3:** MOD by presentation and response to ECV for POP study participants with breech presentation at 36-week scan (*n* = 179).

ECV status	Vaginal	ELCS	EMCS	Total
ECV successful	8	1	3	12
ECV unsuccessful	0	54	18	72
ECV not offered*	1	17	5	23
ECV discussed but declined	1	32	12	45
ECV accepted but not performed†	9	5	11	25
Missing	0	1	1	2
**Total**	19	110	50	179

**Abbreviations:** ECV, external cephalic version; ELCS, elective cesarean section; EMCS, emergency cesarean section; MOD, mode of delivery.

*Eighteen women were contraindicated due to low AFI at screening, 2 for uterine abnormalities, and 3 for other reasons.

†Seventeen babies turned spontaneously, 6 had reduced AFI on the day of the ECV, and 2 went into labour before ECV.

The results from the economic analysis are presented in Table 4. On average, universal ultrasound resulted in an absolute decrease in breech deliveries by 0.39%. It also led to fewer vaginal breech deliveries (absolute decrease by 1.04%) and overall EMCS deliveries (0.72%) than selective ultrasound but increased overall deliveries through ELCS (1.51%). Resulting from the more favourable distribution of MOD, the average risk of mortality fell by 0.0013%. On average, 40 women had to be scanned to identify one previously unsuspected breech presentation (95% Credibility Interval [CrI]: 33 to 49); across England, this would mean that 14,826 (95% CrI: 12,048–17,883) unidentified breech presentations could be avoided annually.

**Table 4:** Simulated cost and MOD distribution for universal ultrasound and no ultrasound.

	Universal ultrasound	Selective ultrasound	Difference (per patient)	Difference (total population)
**Total cost**	2,956.59	2,949.30	7.29	4,268,004
** Screening cost**	20.70	0.43	20.27	11,867,159
** ECV cost**	6.52	2.94	3.57	2,093,048
** Delivery cost**	2,927.78	2,944.31	−16.53	−9,679,396
** Mortality cost**	1.59	1.62	−0.02	−12,806
**Vaginal cephalic**	0.6850	0.6826	0.0024	1,399
**ELCS cephalic**	0.0442	0.0441	0.0001	84
**EMCS cephalic**	0.2321	0.2305	0.0016	918
**VB**	0.0007	0.0110	−0.0104	−6,061
**ELCS breech**	0.0273	0.0123	0.0150	8,774
**EMCS breech**	0.0107	0.0194	−0.0087	−5,115
**Total mortality**	0.000982	0.000995	−0.000013	−7.89
**Total QALY**	24.27615	24.27582	0.000327	191.73

**Abbreviations:** ECV, external cephalic version; ELCS, elective cesarean section; EMCS, emergency cesarean section; MOD, mode of delivery; QALY, quality-adjusted life years; VB, vaginal breech.

Costs (£) are presented per patient, except in column for ‘total population’ (*n* = 585,489).

The expected per person cost of universal ultrasound was £2,957 (95% CrI: £2,922–£2,991), compared to £2,949 (95% CrI: £2,915–£2,984) from selective ultrasound, a cost increase of £7.29 (95% CrI: 2.41–11.61). Across England, this means that universal ultrasound would cost £4.27 million more annually than current practice. The increase stems from higher costs of ultrasound scan (£20.3 per person) and ECV (£3.6 per person) but is partly offset by the lower delivery costs (−£16.5 per person). The distribution of differences in costs between the two strategies is shown as Supporting information, S2 Fig. The simulation shows that universal ultrasound would, on average, increase the number of total ELCS deliveries by 8,858 (95% CrI: 7,662–10,068) but decrease the number of EMCS and vaginal breech deliveries by 4,196 (95% CrI: 2,779–5,603) and 6,061 (95% CrI: 6,617–8,670) per year, respectively.

The long-term health outcomes are presented in Table 4. Nationwide, universal ultrasound would be expected to lower mortality by 7.89 cases annually (95% CrI: 3.71, 12.7). After discounting, this means that universal ultrasound would be expected to yield 192 QALYs annually (95% CrI: 90,308). The cost effectiveness of universal ultrasound depends on the value assigned to these QALYs. The incremental cost effectiveness ratio (ICER) was £23,611 (95% CrI: 8,184, 44,851), which is of borderline cost effectiveness (given NICE’s willingness to pay of £20,000 to £30,000) [38]. The number needed to scan per prevented mortality was 74,204 (95% CrI: 46,124–157,642).

One-way sensitivity analysis showed that the probability parameter with the greatest impact upon the cost effectiveness of universal ultrasound was the prevalence of breech: increasing this parameter by 1 percentage point was associated with a relative reduction of costs for universal ultrasound by £3.07. The results were less sensitive to the ECV success rate; an increase by 1 percentage point led to a relative reduction in the cost of universal ultrasound by £0.12. The most important cost parameter was the unit cost of ultrasound scan; an increase in this parameter by £10 led to a relative increase for universal ultrasound by £9.79 (see Supporting information, S3 Fig). Keeping all other parameters equal, universal ultrasound would be cost effective if ultrasound scanning could be provided for less than £19.80 or £23.10 per mother, for a willingness-to-pay threshold of £20,000 or £30,000, respectively. For universal ultrasound to be cost saving, scans would need to cost less than £12.90 per mother.

## Discussion

In a prospective cohort study of >3,800 women having first pregnancies, a presentation scan at approximately 36 wkGA identified the 4.6% of women who had a foetus presenting by the breech, and for more than half of these, breech presentation had not previously been clinically suspected. The majority of these women were ultimately delivered by planned cesarean section, some experienced labour before their scheduled date and were delivered by EMCS, and a small proportion had a cephalic vaginal delivery following either spontaneous cephalic version or ECV. No woman in the cohort had a vaginal breech delivery or experienced an intrapartum cesarean for undiagnosed breech. The low uptake of vaginal breech birth is likely to reflect the fact that this is a nulliparous population, and it is generally accepted that the risks associated with vaginal breech delivery are lower in women who have had a previous normal birth.

Our economic analysis suggests that a universal late-pregnancy presentation scan would decrease the number of foetal mortalities associated with breech presentation and that this is of borderline cost effectiveness, costing an estimated £23,611 per QALY gained. The key driver of cost effectiveness is the cost of the scan itself. In the absence of a specific national unit cost, we have identified the maximum cost at which it would be cost effective. This is £19.80 per scan to yield an ICER of £20,000 per QALY and £23.10 at £30,000. These unit costs may be possible if assessment of presentation could be performed as part of a routine antenatal visit. Portable ultrasound systems adequate for presentation scans are available at low cost, and a presentation scan is technically quite simple, so the required level of skill could be acquired by a large cadre of midwives. This would result in a small fraction of the costs associated with a trained ultrasonographer performing a scan in a dedicated space using a high-specification machine. If universal ultrasound could be provided for less than £12.90 per scan, the policy would also be cost saving.

Our sensitivity analysis shows that the unit cost of ultrasound scans and the prevalence of breech presentation were by far the biggest determinants of the cost and cost effectiveness of universal ultrasound. The detection rate with abdominal palpation (i.e., for selective ultrasound) is the most important parameter aside from these. By contrast, the costs, attempt, and success rates for ECV have modest impact upon the choice of scanning strategy. It appears that the main short-term cost benefit from late-pregnancy screening lies in the possibility of scheduling ELCSs when breech presentation is detected, rather than turning the baby into a cephalic position.

This analysis may have underestimated the health benefits of universal late-pregnancy ultrasound. In the absence of suitable data on long-term outcomes by MOD and foetal presentation, we made the simplifying assumption that mortality rates were equal for ELCSs and EMCSs. Relaxing this assumption would likely favour universal ultrasound, as this strategy would reduce EMCSs, and these are associated with higher risks of adverse outcomes than ELCSs [41–44]; on top of health benefits, this may also reduce long-term NHS costs. It is also possible that an EMCS for a known breech presentation is less expensive and has better health outcomes than one for which breech is detected intrapartum, although lack of separate data for these two scenarios prevented us from pursuing this analysis further.

Our analysis shows that universal late-pregnancy ultrasound screening would increase total number of cesarean sections. Evidence suggests that cesarean delivery may have long-term consequences on the health of the child (increased risk of asthma and obesity), the mother (reduced risk of pelvic organ prolapse and increased risk of subfertility), and future pregnancies (increased risk of placenta previa and stillbirth) [45,46]. There is no evidence that these are related to the type of the cesarean section (elective versus emergency) [45,46]. Our economic modelling has not been able to capture these complex effects due to the model’s endpoints and the focus on the current pregnancy only. However, accounting for these effects, it seems plausible that universal late-pregnancy ultrasound would be more favourable for mothers than children or future pregnancies.

Our results are also driven by vaginal delivery yielding worse long-term health outcomes than ELCS for breech presentation [1]. However, even though the rate of vaginal breech birth declined after the Term Breech Study, in many cases, the outcomes are not inferior to that of ELCS, and the RCOG guidelines state that vaginal breech delivery may be attempted following careful selection and counselling [1,3,47]. It is hard to assess how an increase in vaginal breech delivery would affect the cost effectiveness of universal ultrasound; while decreased mortality risk from vaginal breech delivery would decrease the importance of knowing the foetal presentation, universal screening would facilitate selection for attempted vaginal breech delivery.

One limitation of this study is that foetal presentation was revealed to all women in the POP study. Consequently, this study cannot say what would have happened without routine screening. However, we felt that it was appropriate to reveal the presentation at the time of the 36-wkGA scan, as there is level 1 evidence that planned cesarean delivery reduces the risk of perinatal morbidity and mortality in the context of breech presentation at term [44]. Another weakness was that the study was being undertaken in a single centre only and that the sample size was too small to avoid substantial parameter uncertainty for rare events. Moreover, less than half of all breech presentations in the POP study were detected by abdominal palpation. It is unclear whether the detection rates were affected by midwives knowing that the women were part of the POP study and, hence, would receive an ultrasound scan at 36 wkGA.

The prevalence of breech presentation in this study (4.6%) appears higher than the 3%–4% that is often reported in literature [1]. However, this study is unique in that it reports the prevalence at the time of ultrasound scanning, approximately 36 wkGA. Taking into account the number of spontaneous reversions to cephalic and that some cases of successful ECV may have turned spontaneously without intervention, our finding is consistent with the literature. The ECV success rate in the POP study was considerably lower than reported elsewhere in the literature; it was even lower than the 32% success rate that has been reported as the threshold level for when ECV is preferred over no intervention at all [48]. This might partly reflect the participants in the POP study; they were older and more likely to be obese than in many previous studies, and the cohort consisted of nulliparous women, who have higher rates of ECV failure than parous women [9,49,50]. It is also possible that the real-world ECV success rate is lower than in the literature due to publication bias. However, sensitivity analysis indicates that the impact from an increased ECV success rate would be modest (an increase in ECV success rate by 10 percentage points lowers the incremental cost of universal ultrasound by £0.91 per patient).

The findings from this study cannot easily be transferred to another health system due to the differences in healthcare costs and antenatal screening routines. Some countries, e.g., France and Germany, already offer a third-trimester routine ultrasound scan. However, these scans are offered prior to 36 wkGA, and as many preterm breech presentations revert spontaneously, it would have limited predictive value for breech at term [51]. Whether screening for breech presentation in lower-income settings is likely to be cost effective largely depends on the coverage of the healthcare system; while screening may be relatively more costly, the benefits from avoiding undiagnosed breech presentation may also be relatively larger.

Whether the findings of this study could be extrapolated beyond nulliparous women is hard to assess. The absence of comparable data on screening sensitivity without universal ultrasound for parous women is an important limitation. The risks associated with breech birth also differ between nulliparous and parous women [52,53]. Compared to nulliparous women, parous women have higher success rates for ECV but also higher risk of spontaneous reversion to breech after 36 wkGA [9,28]. Also, the risks associated with vaginal breech delivery are lower in women who have had a previous vaginal birth [30].

Breech presentation is not the only complication that could be detected through late-pregnancy ultrasound screening. The same ultrasound session could also be used to screen for other indicators of foetal health, such as biometry and signs of growth restriction. Whether also scanning for other complications could increase the benefits from universal ultrasound has been and currently is subject to research [54,55]. Exploring the consequences from such joint screening strategies goes beyond the scope of this paper but has important implications for policy-makers and should therefore be subject to further research.

### Conclusion

This study shows that implementation of universal late-pregnancy ultrasound to assess foetal presentation would virtually eliminate undiagnosed intrapartum breech presentation in nulliparous women. If this procedure could be implemented into routine care, for example, by midwives conducting a routine 36-wkGA appointment and using a portable ultrasound system, it is likely to be cost effective. Such a programme would be expected to reduce the consequences to the child of undiagnosed breech presentation, including morbidity and mortality.

## Supporting information

S1 STROBE checklist STROBE, strengthening the reporting of observational studies in epidemiology. (DOC)

S1 Text Cost input estimation. (DOCX)

S1 Table Input costs and probabilities for the economic model, detailed. (DOCX)

S1 Fig Foetal presentation and ECV status in the POP breech study. ECV, external cephalic version; POPs, Pregnancy Outcome Prediction.
(TIF)

S2 Fig PSA of cost differences between universal ultrasound and selective ultrasound. PSA, Probabilistic Sensitivity Analysis.
(TIFF)

S3 Fig One-way sensitivity analysis of the difference in costs between universal ultrasound and selective ultrasound. (TIFF)

## Abbreviations

AFI: amniotic fluid index
AGA: appropriate for gestational age
CrI: credibility interval
ECV: external cephalic version
ELCS: elective cesarean section
EMCS: emergency cesarean section
FTE: full-time education
HCHS: Hospital and Community Health Services
ICER: incremental cost effectiveness ratio
IMD: Index of Multiple Deprivation
LGA: large for gestational age
MOD: mode of delivery
NHS: National Health Service
NICE: National Institute for Health and Care Excellence
POP: Pregnancy Outcome Prediction
PSA: probabilistic sensitivity analysis
QALY: quality-adjusted life-year
SGA: small for gestational age
wkGA: weeks of gestational age
RCOG: Royal College of Obstetricians and Gynaecologists
SRC: spontaneous reversion to cephalic
STROBE: Strengthening the Reporting of Observational Studies in Epidemiology
