# Supplementary material for: Screening for breech presentation using universal late-pregnancy ultrasonography: A prospective cohort study and cost effectiveness analysis
Source: PLoS Med. 2019 Apr 16;16(4):e1002778. doi: 10.1371/journal.pmed.1002778 (PMC6467368; doi:10.1371/journal.pmed.1002778)
Supplement: S1 Text — (DOCX) [file pmed.1002778.s002.docx]

**Screening for breech presentation using universal late pregnancy ultrasonography: A prospective cohort study and cost-consequences analysis - Appendix.**

David Wastlund^1^, Alexandros A Moraitis^2^, Alison Dacey^2^, Ulla Sovio^2^, Edward CF Wilson^1,3^, Gordon CS Smith^2^

^1^ Cambridge Centre for Health Services Research, Cambridge Institute of Public Health, CB2 0SR Cambridge, UK
^2^ Department of Obstetrics and Gynaecology, University of Cambridge, NIHR Cambridge Comprehensive Biomedical Research Centre, CB2 2SW Cambridge, UK
(AA Moraitis, Clinical Research Fellow; A Dacey, Research Midwife; U Sovio, Senior Research Associate in Applied Medical Statistics; GCS Smith, Professor of Obstetrics and Gynaecology)

^3^ Health Economics Group, Norwich Medical School, University of East Anglia, NR4 7TJ, Norwich, UK

**Correspondence to:**
Prof Gordon CS Smith DSc, Department of Obstetrics and Gynaecology, University of Cambridge, The Rosie Hospital, Cambridge, CB2 2SW, UK.
Tel: 01223 763888/763890; Fax: 01223 763889;
E-mail: [gcss2@cam.ac.uk](mailto:gcss2@cam.ac.uk)

Dr Edward CF Wilson PhD, Health Economics Group, Norwich Medical School, University of East Anglia, Norwich, NR4 7TJ
Tel: 01603 593620
E-mail: [ed.wilson@uea.ac.uk](mailto:ed.wilson@uea.ac.uk)

**S1 Text: Cost input estimation.**

**Mode of Delivery costs:**

The cost per mode of delivery was calculated using the NHS National Schedule of Reference Costs, 2015-16.[1] The costs used were the total HRG’s for each mode of delivery. Multiple costs were available for each mode of delivery, based upon level of complication. We obtained the input value by weighting the unit cost of each complication level by its share of the total procedures.

Since the National Schedule of Reference Costs does not list separate costs for vaginal breech deliveries, we made the simplifying assumption that these costs would have the same ratio to the costs of elective caesarean section as reported by Palencia et al. (2006).[2] For that study, the costs were Ca$7,255 and Ca$8,440 for elective caesarean section and vaginal breech delivery, respectively.

**Ultrasound scan costs:**

There is currently no published cost of an ultrasound scan for assessing fetal presentation only. The NHS reference costs include costs for ‘Ante-Natal Standard Ultrasound scan (NZ21Z)’. However, such scans frequently involve assessment of fetal anatomy and/or biometry and since these require much more time and training to assess than fetal presentation alone, we deemed it inappropriate to use this cost as an estimate for the cost of an ultrasound scan in the context of this analysis. Instead, we theorized that an ultrasound scan for fetal presentation alone could be provided by a midwife in conjunction with a standard antenatal visit in primary care. We estimated the cost of ultrasound scan for fetal presentation using an estimate of the midwife’s time, and the average cost of the basic ultrasonographic equipment needed to assess fetal presentation.

We obtained the cost of the midwife’s time from the Unit Costs of Health and Social Care 2017.[3] NHS staff earnings 2017. On top of the scan itself, time would be needed to make the woman feel comfortable in the process, and to document the results of the scan, we estimated that this would require 7-10 minutes for the average scan. We used the total hourly cost for Band 5 nurses, £36; this cost was consistent with the costs reported for midwives in NHS Staff Earnings 2017.[4] To estimate equipment costs, we used costs for the ACUSON S2000 Ultrasound system. The total cost of this ultrasound machine for the NHS has been estimated to £87,718 in a costing statement by NICE.[5] We assumed that the average machine would be operated 800 to 3000 times annually over the 5-year time horizon. We assumed that room costs would be between £4,500 and £6,000 annually[6], and that rooms would be operated 1,573 hours per year[3].

We simulated the total cost per scan using uniform distributions and 100,000 simulations. The estimated cost per ultrasound scan was £20.7 (95% CI: £11.4, £30.0).

**ECV costs:**

We used the same cost of External Cephalic Version (ECV) as reported by James et al. (2001),[7] updating them for inflation. James et al. (2001) reported two different costs; we used the estimate for high staff costs: £193.3. To convert to 2017’s price level, we used the Hospital & Community Health Services (HCHS) inflation index: compared to baseline, the index was 302.3 for year 2017,[3] and 196.5 for year 2001.[8] The resulting cost per ECV was £297.4.

**References**

1. Department of Health. NHS Reference Costs 2015-16, 2016.

2. Palencia R, Gafni A, Hannah ME, et al. The costs of planned cesarean versus planned vaginal birth in the Term Breech Trial. *CMAJ* 2006;174(8):1109-13.

3. Curtis L, Burns A. Unit Costs of Health and Social Care 2017: Personal Social Services Research Unit, 2017.

4. NHS Digital. NHS Staff Earnings - Estimates ot September 2017, Provisional Statistics, 2017.

5. National Institute of Clinical Excellence. Implementing the NICE guidance on Virtual Touch Quantification to diagnose and monitor liver fibrosis in chronic hepatitis B and C (MTG27), 2015.

6. NHS Purchasing and Supply Agency. Cost-effectiveness of ultrasound elastography in the assessment of liver fibrosis, 2009.

7. James M, Kevin H, B. R, et al. A decision analytical cost analysis of offering ECV in a UK district general hospital. *BMC Health Services Research* 2001;1(6).

8. Curtis L. Unit Costs of health and Social Care 2008. Cornwallis Building, The University of Kent, Canterbury, Kent CT2 7NF: Personal Social Services Research Unit, 2008.

9. Ben-Meir A, Elram T, Tsafrir A, et al. The incidence of spontaneous version after failed external cephalic version. *American journal of obstetrics and gynecology* 2007;196(2):157.e1-3.

10. Leung WC, Pun TC, Wong WM. Undiagnosed breech revisited. *BJOG: an international journal of obstetrics and gynaecology* 1999;106(7):638-41.

**Figure legends**

**Figure S1:** Fetal presentation and ECV status in the POP breech study

*Schedule of fetal presentation and ECV status in the POP breech study. The mode of delivery is not presented to prevent identification of participants.
ECV = External cephalic version*

**Figure S2:** Probabilistic Sensitivity Analysis (PSA) of cost differences between universal ultrasound and selective ultrasound

*Distribution of differences in costs between universal ultrasound and no ultrasound. The horizontal axis shows the difference in costs between the two scenarios; a positive value means that universal ultrasound is more costly than no ultrasound. The vertical axis shows the share of simulations that fell within each cost difference interval.*

**Figure S3:** One-way sensitivity analysis of the difference in costs between universal ultrasound and selective ultrasound

*One-way sensitivity analysis of how a change in input values for each parameter affects the difference in per patient costs between the two screening strategies. Probabilities (top chart) are changed by 1 percentage point; costs (bottom chart) are changed by £10. Cost differences are shown on the horizontal axis; negative values means a difference in favour of universal ultrasound, positive values favour no ultrasound. Decreases in input values are shown in white and increases in grey.
CV = Cephalic vaginal delivery; ELCS = Elective caesarean section; EMCS = Emergency caesarean section; VB = Vaginal breech delivery; ECV = External cephalic version; SRC = Spontaneous reversion to cephalic; APDL = Abdominal palpation detection likelihood*
