# Supplementary material for: Screening for breech presentation using universal late-pregnancy ultrasonography: A prospective cohort study and cost effectiveness analysis
Source: PLoS Med. 2019 Apr 16;16(4):e1002778. doi: 10.1371/journal.pmed.1002778 (PMC6467368; doi:10.1371/journal.pmed.1002778)
Supplement: S1 Table — (DOCX) [file pmed.1002778.s003.docx]

**S1 Table: Input costs and probabilities for the economic model, detailed**

| **Costs** | **Mean** | **95% CI** | **Distribution** | **Source** | |
| --- | --- | --- | --- | --- | --- |
| Ultrasound scanning | 20.7 | 18.7, 22.7 | ~Uniform(18.6, 22.8) | Expert opinion * | |
| ECV | 297.4 | 269.2, 325.6 | ~Uniform(267.7, 327.1) | James et al.[7] † | |
| CV delivery | 2297.3 | 2079.1, 2515.5 | ~Uniform(2067.6, 2527.0) | NHS Reference costs 2015-16[1] ‡ | |
| Elective caesarean delivery | 3438.1 | 3111.5, 3764.7 | ~Uniform(3094.3, 3781.9) | NHS Reference costs 2015-16[1] ‡ | |
| Emergency caesarean delivery | 4553.4 | 4120.9, 4985.9 | ~Uniform(4098.1, 5008.7) | NHS Reference costs 2015-16[1] ‡ | |
| VB delivery | 3999.7 | 3619.7, 4379.7 | ~Uniform(3599.7, 4399.7) | Expert opinion * | |
| **Probabilities** | **Mean** | **95% CI** | **Distribution** | **Node** | **Source** |
| Breech prevalence at ~36wkGA | 0.046 | 0.040, 0.053 | ~Beta(179, 3700) | A1 & B1 | POP Study ** |
| ECV attempted | 0.475 | 0.402, 0.548 | ~Beta(84, 93) | C1 | POP Study ** |
| Detection without ultrasound | 0.451 | 0.379, 0.525 | ~Beta(79, 96) | B3 | POP Study ** |
| Successful ECV | 0.143 | 0.077, 0.225 | ~Beta(12, 72) | C2 | POP Study ** |
| SRC (ECV not attempted) | 0.226 | 0.147, 0.316 | ~Beta(21, 72) | C3 | POP Study ** |
| SRB | 0.083 | 0.002, 0.285 | ~Beta(1, 11) | C4 | POP Study ** |
| SRC (failed ECV) | 0.023 | 0.005, 0.055 | ~Beta(3, 127) | C5 | Ben Meir et al.[9] § |
| **Mode of delivery** | **Distribution** | | | **Node** | **Source** |
| No breech †† | ~Dirichlet(2813, 141, 735) | | | A2 & B2 | POP Study ** |
| Cephalic (successful ECV) †† | ~Dirichlet(8, 0, 3) | | | C8 | POP Study ** |
| Cephalic (spontaneous reversion) †† | ~Dirichlet(11, 1, 9) | | | C6 & C10 | POP Study ** |
| Breech (ECV not attempted) ‡‡ | ~Dirichlet(52, 20, 0) | | | C7 | POP Study ** |
| Breech (Unsuccessful ECV) ‡‡ | ~Dirichlet(54, 18, 0) | | | C11 | POP Study ** |
| Breech (spontaneous reversion) ‡‡ | ~Dirichlet(0, 15, 11) | | | C9 | Leung et al.[10] |
| Undetected breech ‡‡ | ~Dirichlet(0, 15, 11) | | | B4 | Leung et al.[10] |

Costs given per unit/episode. For probabilities, Alpha represent case of event and Beta case of no event. Mode of delivery shows input values for Dirichlet distribution. Node refers to the chance nodes in Fig 1.
CV = Cephalic Vaginal; ELCS = Elective caesarean section; EMCS = Emergency caesarean section; IDR = Incidental detection rate; SRB = Spontaneous reversion to breech; SRC = Spontaneous reversion to cephalic; VB = Vaginal breech
* Details on how this value was estimated is provided as supporting information, S1 Text.
† Cost for ECV (high staff cost), converted to 2017 price level using the Hospital & Community Health Services (HCHS) index.[3]
‡ Weighted average of all complication levels (Total HRG’s)

†† Distribution is for cephalic vaginal delivery, elective CS delivery, and emergency CS delivery, respectively

‡‡ Distribution is for elective CS delivery, emergency CS delivery, and vaginal breech delivery, respectively

§ Due to the small sample size for these parameters in the POP study, the model used inputs for mode of delivery for undetected breech instead.
