# Supplementary figures and images for: Screening for breech presentation using universal late-pregnancy ultrasonography: A prospective cohort study and cost effectiveness analysis
Source: PLoS Med. 2019 Apr 16;16(4):e1002778. doi: 10.1371/journal.pmed.1002778 (PMC6467368; doi:10.1371/journal.pmed.1002778)

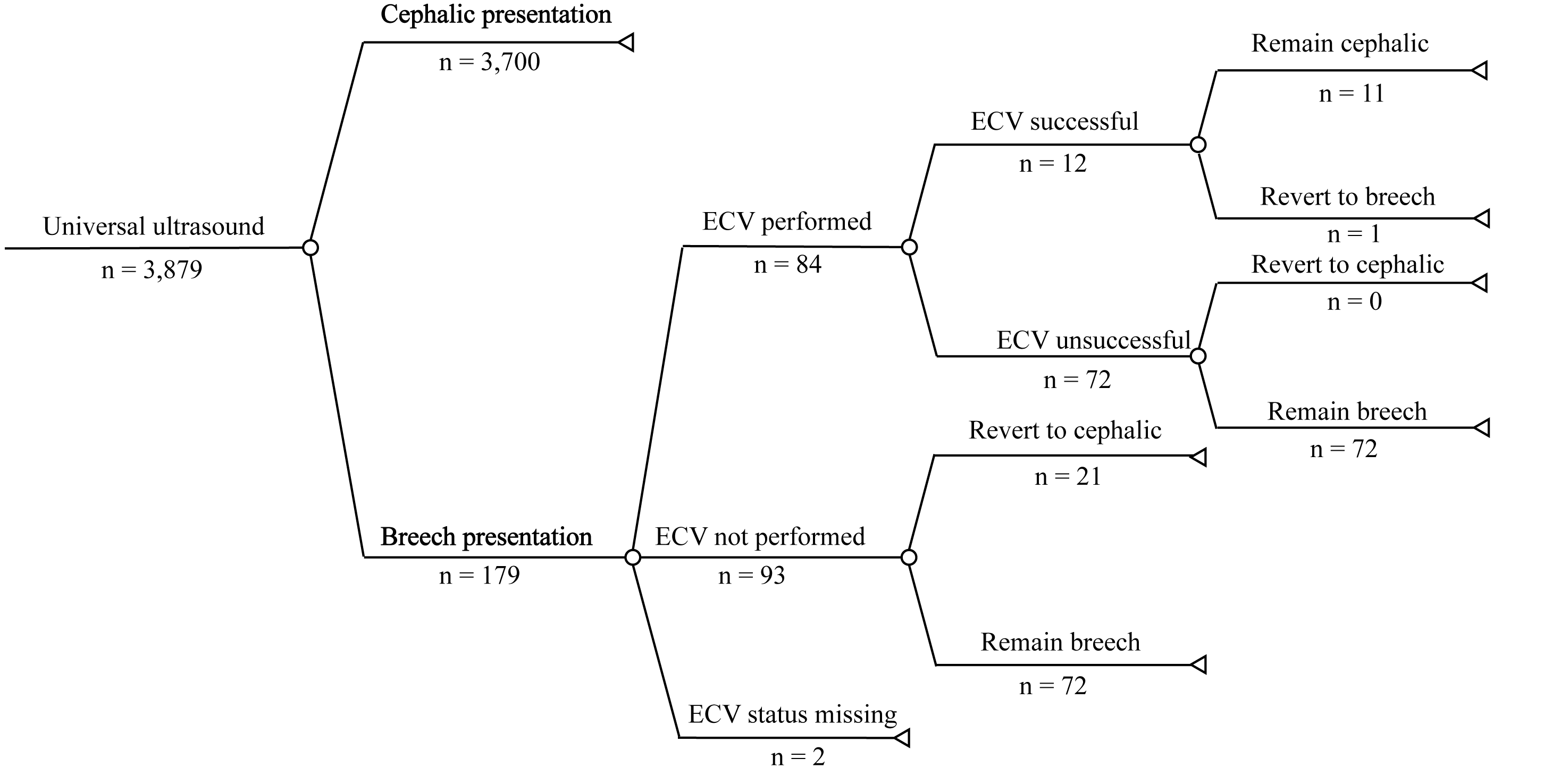

Supplement: S1 Fig — ECV, external cephalic version; POPs, Pregnancy Outcome Prediction. (TIF) [file pmed.1002778.s004.tif]

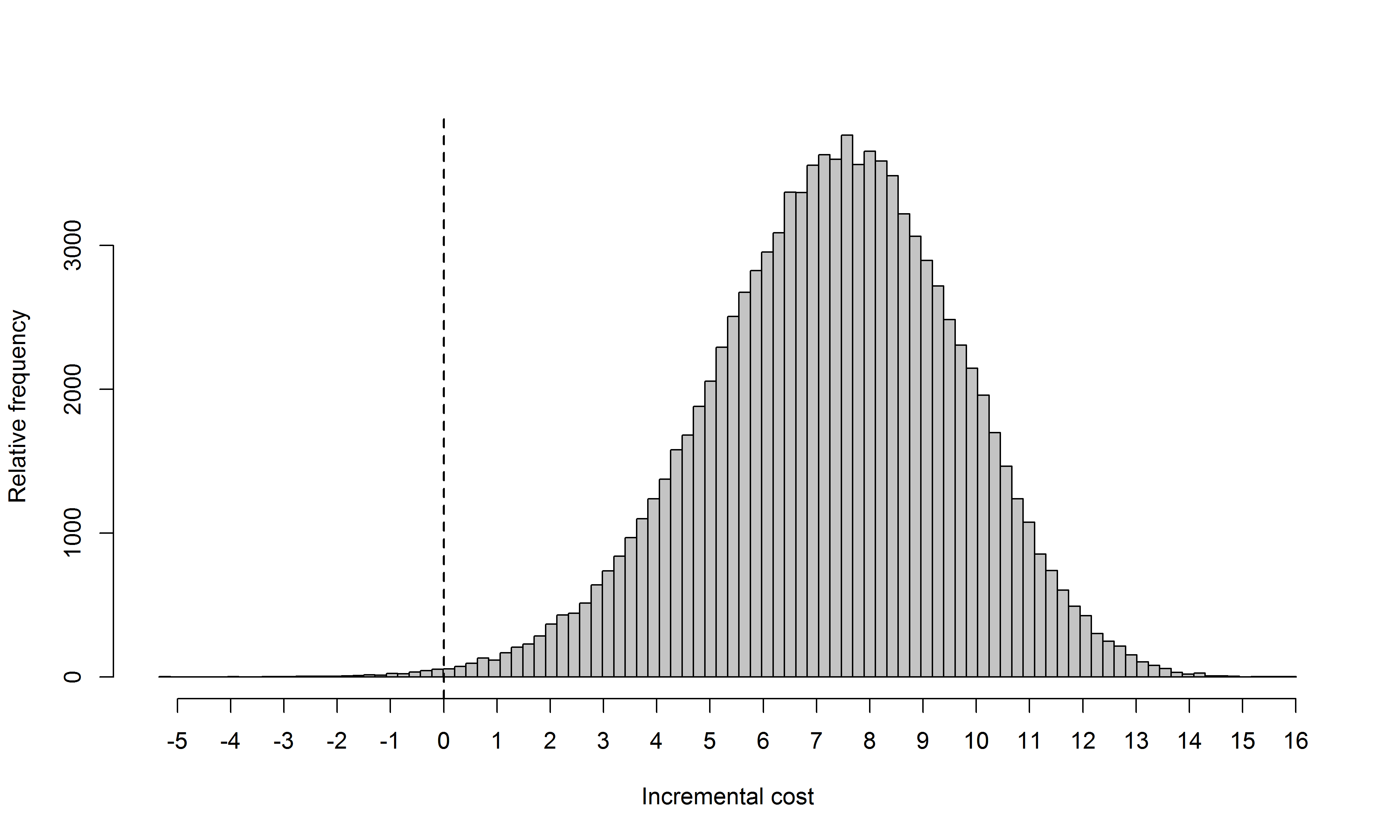

Supplement: S2 Fig — PSA, Probabilistic Sensitivity Analysis. (TIFF) [file pmed.1002778.s005.tiff]

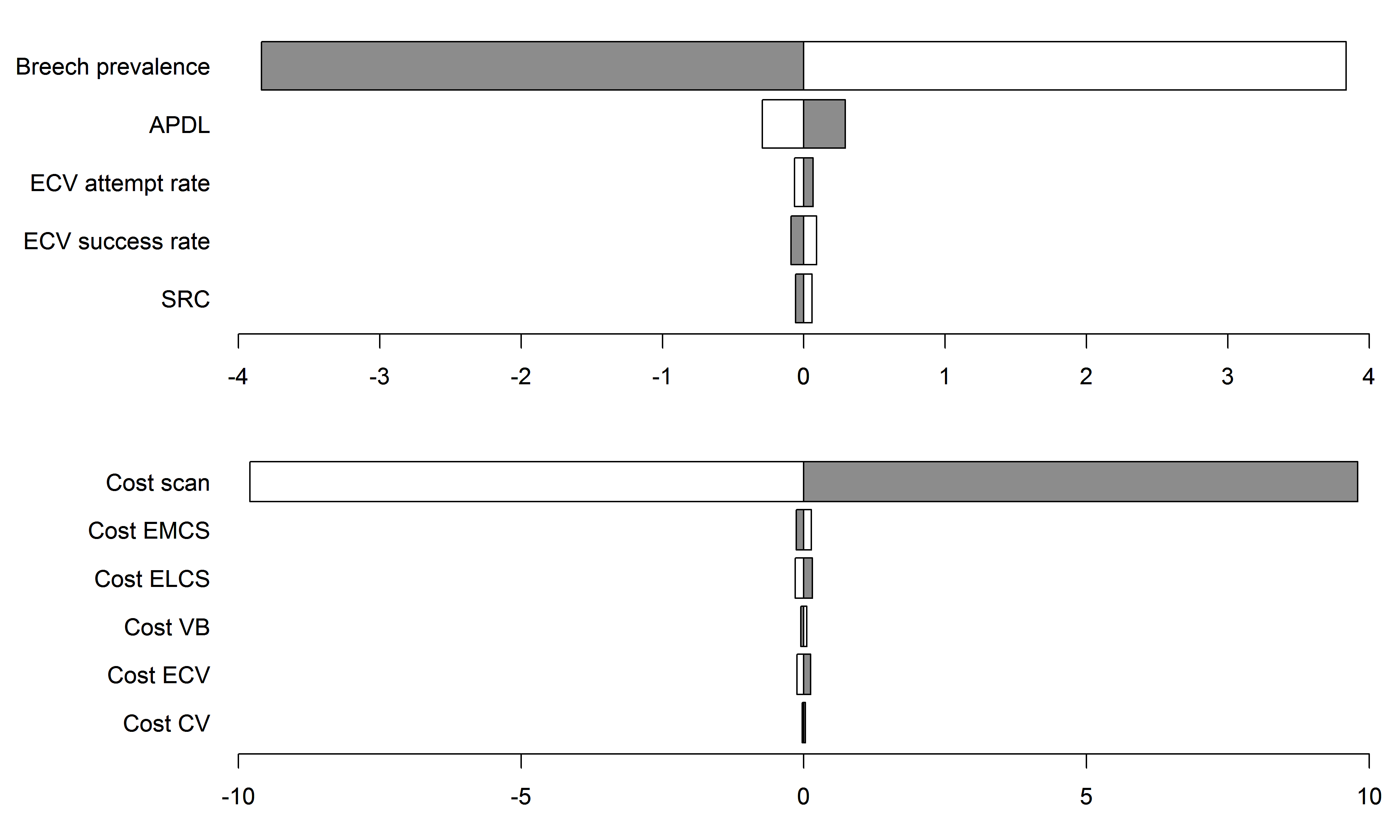

Supplement: S3 Fig — (TIFF) [file pmed.1002778.s006.tiff]
